# Progesterone Attenuates Stress-Induced NLRP3 Inflammasome Activation and Enhances Autophagy Following Ischemic Brain Injury

**DOI:** 10.3390/ijms21113740

**Published:** 2020-05-26

**Authors:** Claudia Espinosa-Garcia, Fahim Atif, Seema Yousuf, Iqbal Sayeed, Gretchen N. Neigh, Donald G. Stein

**Affiliations:** 1 Department of Emergency Medicine, Emory University, Atlanta, GA 30322, USA; fatif@emory.edu (F.A.); syousu2@emory.edu (S.Y.); isayeed@emory.edu (I.S.); dstei04@emory.edu (D.G.S.); 2 Department of Anatomy and Neurobiology, Virginia Commonwealth University, Richmond, VA 23298, USA; gretchen.mccandless@vcuhealth.org

**Keywords:** stress, microglial priming, alarmins, HMGB1, NLRP3 inflammasome, autophagy, cerebral ischemia, progesterone

## Abstract

NOD-like receptor pyrin domain containing 3 (NLRP3) inflammasome inhibition and autophagy induction attenuate inflammation and improve outcome in rodent models of cerebral ischemia. However, the impact of chronic stress on NLRP3 inflammasome and autophagic response to ischemia remains unknown. Progesterone (PROG), a neuroprotective steroid, shows promise in reducing excessive inflammation associated with poor outcome in ischemic brain injury patients with comorbid conditions, including elevated stress. Stress primes microglia, mainly by the release of alarmins such as high-mobility group box-1 (HMGB1). HMGB1 activates the NLRP3 inflammasome, resulting in pro-inflammatory interleukin (IL)-1β production. In experiment 1, adult male Sprague-Dawley rats were exposed to social defeat stress for 8 days and then subjected to global ischemia by the 4-vessel occlusion model, a clinically relevant brain injury associated with cardiac arrest. PROG was administered 2 and 6 h after occlusion and then daily for 7 days. Animals were killed at 7 or 14 days post-ischemia. Here, we show that stress and global ischemia exert a synergistic effect in HMGB1 release, resulting in exacerbation of NLRP3 inflammasome activation and autophagy impairment in the hippocampus of ischemic animals. In experiment 2, an *in vitro* inflammasome assay, primary microglia isolated from neonatal brain tissue, were primed with lipopolysaccharide (LPS) and stimulated with adenosine triphosphate (ATP), displaying impaired autophagy and increased IL-1β production. In experiment 3, hippocampal microglia isolated from stressed and unstressed animals, were stimulated *ex vivo* with LPS, exhibiting similar changes than primary microglia. Treatment with PROG reduced HMGB1 release and NLRP3 inflammasome activation, and enhanced autophagy in stressed and unstressed ischemic animals. Pre-treatment with an autophagy inhibitor blocked Progesterone’s (PROG’s) beneficial effects in microglia. Our data suggest that modulation of microglial priming is one of the molecular mechanisms by which PROG ameliorates ischemic brain injury under stressful conditions.

## 1. Introduction

Chronic stress has been implicated in increasing the risk of cardiovascular events, including cardiac arrest and worsening outcomes [1,2,3]. Under conditions of stress, microglia—the principal immune effectors in the CNS—become hypersensitive or “primed” and overreact to a secondary insult by producing factors that are highly inflammatory and neurotoxic [4,5]. Increasing evidence indicates that microglial overactivation leads to exacerbated neuronal damage and excessive brain inflammation in stressed ischemic animals [6,7,8,9,10,11]. From a translational perspective, reducing the neurotoxic potential of primed microglia has important implications for the treatment of ischemic brain injury patients with elevated stress.

Among other factors, stress-induced microglial priming is mediated by an alarmin called high-mobility group box-1 (HMGB1) [12]. HMGB1 is a ubiquitous nuclear protein that can be released in the brain either by inflammatory cells in response to stress [13,14] or by necrotic cells following ischemic brain injury [15,16,17,18]. Several studies have reported elevated HMGB1 levels in the serum/plasma of resuscitated patients [19,20]. Once released into the extracellular space, HMGB1 binds to its receptors and triggers the activation of the inflammasomes [21].

The NLRP3 inflammasome is a multiprotein complex consisting of a sensor molecule, NLRP3, which interacts with the adaptor protein ASC; and caspase-1, a protease whose activation induces cleavage and release of pro-inflammatory cytokines such as IL-1β [22]. NLRP3 inflammasome activation requires a priming step and a second activation step [23]. It is noteworthy that this inflammasome is primed by stress [13,24] and activated following ischemic injury [25]. Inhibiting NLRP3 activation reduces the inflammation associated with poor outcomes in rodent models of brain injury associated with cardiac arrest [26,27].

In response to an injury, the NLRP3 inflammasome can be regulated by autophagy, an intracellular homeostatic recycling process that eliminates damaged organelles and toxic agents, including inflammasomes [28]. Emerging research shows that autophagy modulates microglial inflammatory response via NLRP3 degradation [29], and autophagy impairment worsens neuroinflammation [30,31] and ischemic injury [32]. The pharmacological induction of autophagy inhibits inflammation and improves outcome in rodent models of cardiac arrest [33,34]. An understanding of how stress alters NLRP3 activation and autophagy, thus priming the microglial inflammatory response to ischemia, is needed. Without such information, the potential of NLRP3 inflammasome inhibition and autophagy induction as therapeutic approaches for ischemic injury associated with cardiac arrest and worsened by comorbid stress will likely remain elusive.

It is well established that progesterone (PROG), a neuroprotective steroid, reduces pro-inflammatory cytokine expression and regulates microglial activation and polarization following ischemic injury [9,25,35,36,37,38,39,40,41,42,43,44,45,46]. However, the molecular mechanisms underlying PROG’s effects on the aggravated inflammatory response in the ischemic brain potentiated by comorbid stress have not been fully elucidated.

We recently showed that stress potentiates microglial activation, dysregulates M1/M2 polarization, and worsens the inflammatory environment and neuronal loss in the ischemic hippocampus and that post-ischemic treatment with PROG attenuates this inflammatory response and ameliorates outcome [9]. In the current study, we hypothesized that PROG will modulate stress-induced microglial priming by reducing alarmin HMGB1 release and NLRP3 inflammasome activation while enhancing autophagy (1) in primary microglia after NLRP3 inflammasome activation *in vitro*; (2) in stress-primed hippocampal microglia stimulated with LPS *ex vivo*; and (3) in a rodent model of global ischemia combined with social defeat stress.

## 2. Results

### 2.1. Stress Worsens HMGB1 Release Induced by Global Ischemia

**Figure 1:**
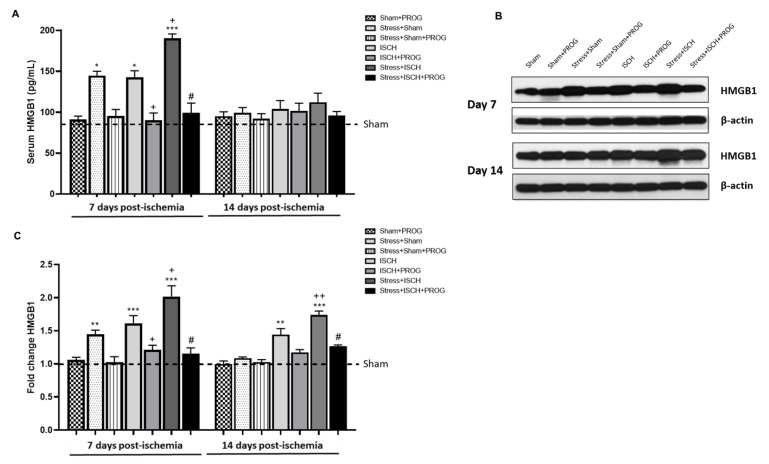
Effects of stress and progesterone on HMGB1 release induced by global ischemia. (**A**) Levels of HMGB1 in serum [ANOVA F(15, 80) = 11.26, *p* < 0.0001], (**B**) representative blots and (**C**) densitometry data for HMGB1 in the hippocampus [ANOVA F(15, 80) = 18.23, *p* < 0.0001], obtained from ischemic animals at 7 and 14 days after global ischemia, or from sham animals. Data are expressed as mean ± SEM. N = 6/group. * *p* < 0.01, ** *p* < 0.001, *** *p* < 0.001 vs. Sham; ^+^ *p* < 0.05, ^++^ *p* < 0.01, ^+++^ *p* < 0.001 vs. ISCH; and ^#^ *p* < 0.001 vs. STRESS+ISCH.

To determine whether social defeat stress synergizes with global ischemia to worsen HMGB1 release and whether PROG treatment can attenuate such release, we measured serum and hippocampal levels of HMGB1 on days 7 and 14 after global ischemia (Figure 1).

At 7 days post-ischemia, serum (Figure 1A) and hippocampal (Figure 1B,C) HMGB1 levels were equally elevated in Stress+Sham and ISCH groups compared to Sham controls. The Stress+ISCH group showed higher serum and hippocampal HMGB1 levels in comparison with unstressed ischemic animals (Figure 1A–C), suggesting a synergistic effect between stress and ischemia on HMGB1 release. At 14 days post-ischemia, serum HMGB1 returned to basal levels (Figure 1A), while HMGB1 levels remained elevated in the hippocampus of stressed and unstressed ischemic animals (Figure 1B,C). Treatment with PROG reduced peripheral and hippocampal HMGB1 release induced by global ischemia, which was worsened by comorbid stress (Figure 1A–C).

### 2.2. Activated Microglia Are the Main Cellular Source of HMGB1 Release

**Figure 2:**
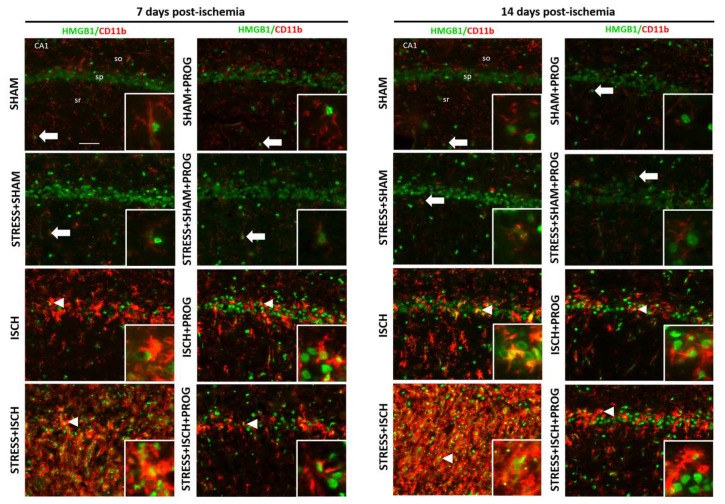
Effects of stress and progesterone on HMGB1 distribution in activated microglia. Representative immunofluorescence merged images showing the distribution of HMGB1 (green) and CD11b positive microglia (red) in the hippocampal CA1 region at 7 and 14 days after global ischemia, or from sham animals. White arrows indicate HMGB1 located in the nuclei of surveillant microglia in sham animals; white arrowheads indicate HMGB1 translocated from the nucleus to the cytoplasm of activated microglia in ischemic animals. Right squares show enlargements of selected cells. so, *stratum oriens*; sp., *stratum pyramidale*; sr, *stratum radiatum*. Scale bar: 50 μm.

Based on our findings showing that stress worsens HMGB1 release in the ischemic hippocampus, we further examined whether stress alters the cellular source of this alarmin on days 7 and 14 after global ischemia. We visualized HMGB1 in neurons, astrocytes, and microglia by double immunofluorescence with cell markers NeuN, GFAP, and CD11b, respectively. In Sham animals, we observed that HMGB1 was localized in the nuclei of neurons (Supplementary Figure S1), astrocytes (Supplementary Figure S2), and surveillant microglia (Figure 2). In contrast, in ischemic animals, HMGB1 was remarkably translocated from the nucleus to the cytoplasm in activated microglia (Figure 2) but remained in the nuclei of surviving neurons and reactive astrocytes (Supplementary Figures S1 and S2) at 7 and 14 days post-ischemia. This neurotoxic inflammatory response was potentiated in microglia from the Stress+ISCH group compared to the ischemia alone group (Figure 2). Our data can be interpreted to indicate that activated microglia were the main cellular source of HMGB1 release in unstressed and stressed ischemic animals at later stages of inflammation. Treatment with PROG decreased HMGB1 cytoplasmic translocation in activated microglia in both unstressed and stressed ischemic animals at 7 and 14 days after global ischemia (Figure 2).

### 2.3. Stress Exacerbates NLRP3 Inflammasome Activation in the Ischemic Hippocampus

**Figure 3:**
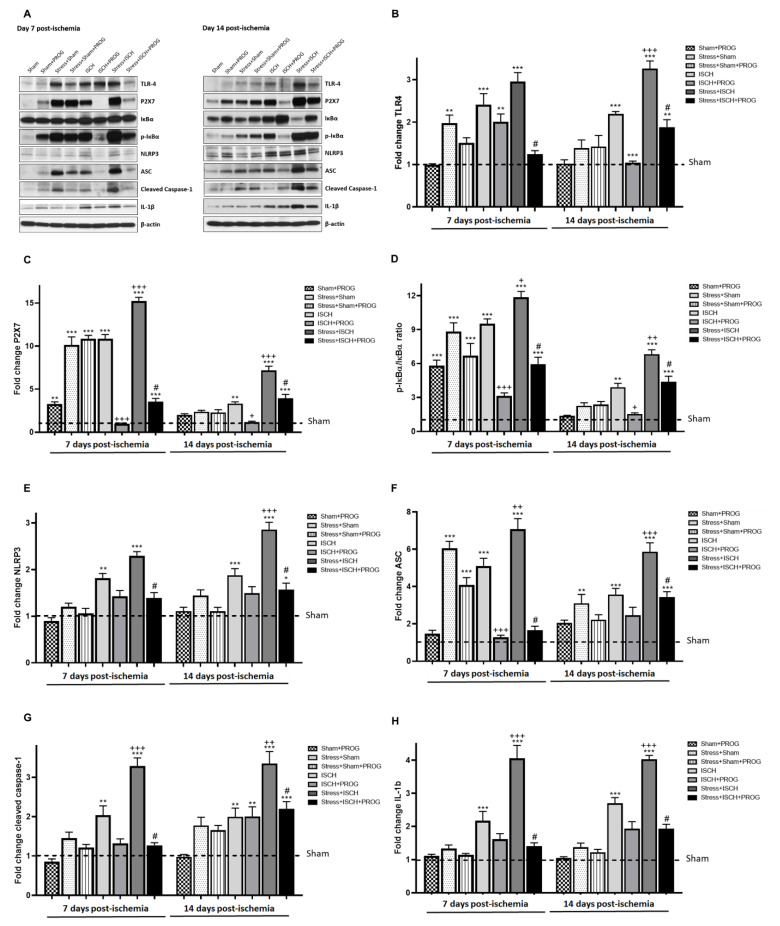
Effects of stress and progesterone on the NLRP3 inflammasome signaling pathway activated by global ischemia. Representative blots (**A**) and densitometry data for TLR4 (**B**, ANOVA F(15, 80) = 21.22, *p* < 0.0001), P2X7 (**C**, ANOVA F(15, 80) = 140.7, *p* < 0.0001), IκBα (**D**, ANOVA F(15, 80) = 50.26, *p* < 0.0001), NLRP3 (**E**, ANOVA F(15, 80) = 24.47, *p* < 0.0001), ASC (**F**, ANOVA F(15, 80) = 33.84, *p* < 0.0001), cleaved caspase-1 (**G**, ANOVA F(15, 80) = 20.56, *p* < 0.0001), IL-1β (**H**, ANOVA F(15, 80) = 38.20, *p* < 0.0001) in the hippocampus obtained from ischemic animals at 7 and 14 days after global ischemia, or from sham animals. Data are expressed as mean ± SEM. N = 6/group. * *p* < 0.01, ** *p* < 0.001, *** *p* < 0.001 vs. Sham; ^+^ *p* < 0.05, ^++^ *p* < 0.01, ^+++^ *p* < 0.001 vs. ISCH; ^#^ *p* < 0.001 vs. STRESS+ISCH.

To determine whether stress-induced HMGB1 release primes, the NLRP3 inflammasome in rats subjected to global ischemia, and whether PROG treatment modulates stress priming, we investigated the NLRP3 inflammasome signaling pathway in the hippocampus on days 7 and 14 after global ischemia (Figure 3).

Stress alone increased the expression of TLR4 (Figure 3A,B), P2X7 (Figure 3A,C), ASC (Figure 3A,E), phosphorylation of NF-κB inhibitor (p-IκBα/IκBα ratio; Figure 3A,F), and cleaved caspase-1 (Figure 3A,G) without changes in IL-1β (Figure 3A,H) in the hippocampus. At day 7, global ischemia-induced NLRP3 inflammasome activation (Figure 3A–E), as evidenced by an increase in the expression of NLRP3, and IL-1β (Figure 3A,H) in the hippocampus compared to Sham controls. It is important to note that TLR4, P2X7, NLRP3, ASC, cleaved caspase-1, and IL-1β remained elevated at day 14 post-ischemia. The current results confirmed previous studies showing that stress alone primed the NLRP3 inflammasome and that global ischemia activated its signaling pathway. Accordingly, stressed ischemic animals showed higher expression of TLR4 (Figure 3A,B), P2X7 (Figure 3A,C), p-IκBα/IκBα ratio (Figure 3A,D), NLRP3 (Figure 3A,E), ASC (Figure 3A,F), cleaved caspase-1 (Figure 3A,G), and IL-1β (Figure 3A,H) in comparison with their unstressed ischemic counterparts. Treatment with PROG reduced NLRP3 inflammasome pathway activation in both unstressed and stressed ischemic animals at 7 and 14 days after global ischemia (Figure 3A–H).

### 2.4. Stress Impairs Autophagy Following Global Ischemia

**Figure 4:**
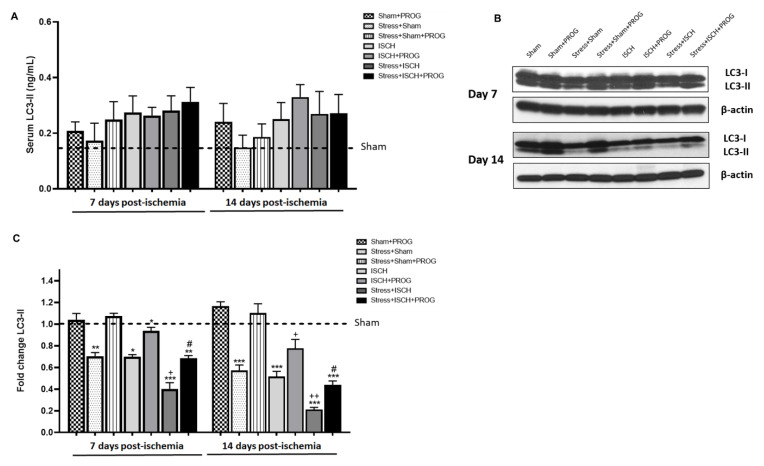
Effects of stress and progesterone on autophagy following global ischemia. (**A**) Levels of LC3-II in serum [ANOVA F(15, 80) = 0.8303, *p* < 0.6417], (**B**) representative blots and (**C**) densitometry data for LC3-II in the hippocampus [ANOVA F(15, 80) = 40.60, *p* < 0.0001], obtained from ischemic animals at 7 and 14 days after global ischemia, or from sham animals. Data are expressed as mean ± SEM. N = 6/group. * *p* < 0.01, ** *p* < 0.001, *** *p* < 0.001 vs. Sham; ^+^ *p* < 0.05, ^++^ *p* < 0.01, ^+++^ *p* < 0.001 vs. ISCH; and ^#^ *p* < 0.001 vs. STRESS+ISCH.

To determine whether social defeat stress impairs autophagy in rats subjected to global ischemia and whether PROG treatment ameliorates this effect, we measured serum and hippocampal levels of LC3-II, an autophagic flux marker that reflects the number of autophagic structures called autophagosomes [47], on days 7 and 14 after global ischemia (Figure 4).

No differences were observed in serum LC3-II levels at either 7 or 14 days post-ischemia (Figure 4A). In contrast, at day 7, hippocampal LC3-II levels were reduced in Stress+Sham and ISCH groups compared to Sham controls and remained low at day 14 (Figure 4B,C). Stressed ischemic animals showed lower hippocampal LC3-II levels than their unstressed ischemic counterparts (Figure 4B,C). Treatment with PROG ameliorated hippocampal autophagy impairment induced by global ischemia and worsened by comorbid stress (Figure 4B,C).

### 2.5. NLRP3 Inflammasome Activation In Vitro Compromises Autophagy in Microglia

**Figure 5:**
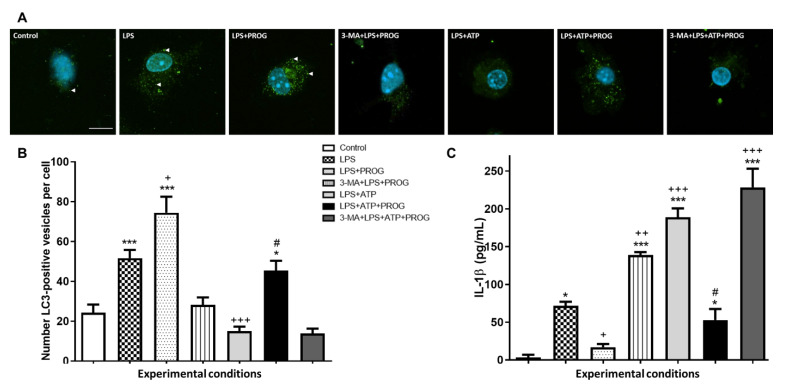
Inflammasome activation *in vitro* compromises autophagy in microglia. Primary cultured microglia were stimulated with LPS (1 µg/mL), followed by ATP (5 mM), and concomitantly treated with PROG (20 µM). Pre-treatment with 3-MA (5 mM) was used to inhibit autophagy. (**A**) Representative immunofluorescence for LC3-positive vesicles (white arrowheads) in microglia isolated from neonatal mice. (**B**) Average number of LC3-positive vesicles per cell in each condition [ANOVA F(6, 159) = 18.63, *p* < 0.0001]. (**C**) IL-1β levels in the culture supernatant, as measured by ELISA [ANOVA F(6, 14) = 83.90, *p* < 0.0001]. Scale bar: 10 μm. Data are expressed as mean±SEM. N = 3 replications. * *p* < 0.05, *** *p* < 0.001 vs. Control; ^+^ *p* < 0.05, ^++^ *p* < 0.01, ^+++^ *p* < 0.001 vs. LPS; and ^#^ *p* < 0.001 vs. LPS+ATP.

To investigate the effects of NLRP3 inflammasome activation on autophagy in microglia, we used an *in vitro* inflammasome assay that involved priming with LPS, followed by stimulation with ATP. Additionally, cells were concomitantly treated with PROG, and autophagy was inhibited by pre-treatment with 3-MA (Figure 5).

As expected [48], stimulation with LPS alone increased the number of LC3-positive vesicles in microglia (Figure 5A,B), as well as IL-1β levels compared to control (Figure 5C). Stimulation with LPS followed by ATP resulted in a lower number of LC3-positive vesicles (Figure 5A,B) and higher IL-1β levels (Figure 5C) compared to LPS alone, indicating that NLRP3 inflammasome activation *in vitro* worsened IL-1β production and compromised microglial autophagy. Treatment with PROG increased the number of LC3-positive vesicles (Figure 5A,C) and reduced IL-1β levels (Figure 5C) in both LPS-alone and LPS+ATP groups. Pharmacological inhibition of autophagy with 3-MA blocked PROG’s pro-autophagic effects and aggravated microglial inflammatory response *in vitro*.

### 2.6. Stress-Primed Microglia Ex Vivo Show Elevated IL-1β Production and Impaired Autophagy

To illuminate the molecular effects of stress in microglia, we adapted a well-established method of “neuroinflammatory priming to stress” isolation of hippocampal microglia from male adult rats that have experienced prior stress, causing microglia to exhibit a potentiated response to a secondary insult *ex vivo* [49].

**Figure 6:**
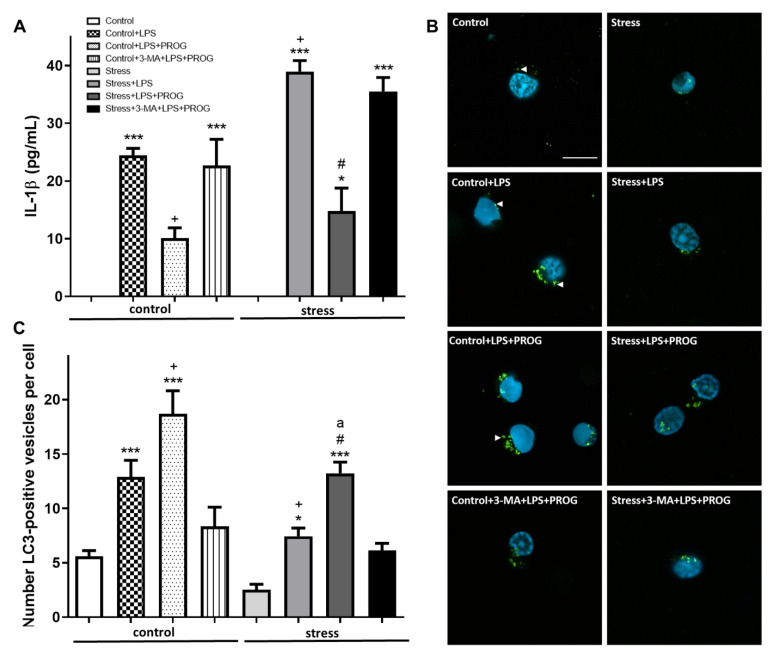
Autophagy impairment in stress-primed microglia. Hippocampal microglia were isolated from male adult rats that have experienced prior stress, or from unstressed controls. Then, cells were stimulated *ex vivo* with LPS (10 ng/mL) and concomitantly treated with PROG (20 µM). Pre-treatment with 3-MA (5 mM) was used to inhibit autophagy. (**A**) IL-1β levels in the culture supernatant, as measured by ELISA [ANOVA F(7, 16) = 34.51, *p* < 0.0001]. (**B**) Representative immunofluorescence for LC3-positive vesicles (white arrowheads) in microglia isolated from unstressed controls (left panel) and stressed animals (right panel). (**C**) Average number of LC3-positive vesicles per cell in each condition [ANOVA F(7, 230) = 21.08, *p* < 0.0001]. Scale bar: 10 μm. Data are expressed as mean ± SEM. N = 3 replications. * *p* < 0.05, *** *p* < 0.001, vs. Control; ^+^ *p* < 0.05 vs. Control+LPS; ^#^ *p* < 0.01 vs. Stress+LPS; and ^a^ *p* < 0.05 vs. control+LPS+PROG.

We confirmed that stress-primed microglia, when exposed to LPS *ex vivo*, produced more IL-1β compared to microglia isolated from unstressed control animals (Figure 6A). We also found that stress-primed microglia showed fewer LC3-positive vesicles compared to controls (Figure 6B,C). These results were consistent with the idea that microglial autophagy can be impaired by stress. Treatment with PROG reduced IL-1β levels (Figure 6A) and enhanced autophagy in both control+LPS and stress+LPS groups (Figure 6B,C). The autophagy inhibitor 3-MA blocked PROG’s proautophagic effects in microglia *ex vivo*.

## 3. Discussion

Numerous studies show that prior exposure to chronic stress, either social isolation, restrain stress, or social defeat worsens global ischemia-induced neuronal death and microglial activation [6,7,8,9,10,11]. However, the molecular and cellular mechanisms underlying the contributions of comorbid stress to worsened cardiac arrest outcomes are not fully understood. The present study is an extension of our previous work [9], showing that comorbid stress exacerbates the inflammatory response, particularly microglial activation and M1/M2 polarization in a rat model of global ischemia. This series of events worsen ischemic injury, but treatment with PROG reduces inflammation and neuronal loss.

### 3.1. Effects of Stress Prior to Ischemia on HMGB1 Release

We can now confirm previous studies showing that the alarmin HMGB1 is released in response to stress [13,14] and ischemia [15,16,17,18,20]. Particularly, Shi et al. [20] demonstrated that serum HMGB1 expression levels were increased in patients at 7 days and rats at 24 h after cardiac arrest. We also found that social defeat stress combined with global ischemia acted in synergy to increase serum and hippocampal HMGB1 levels, which then remained elevated in the hippocampus at 14 days post-ischemia. As we previously reported [9], this synergistic effect on HMGB1 release might have contributed to worsening inflammation and neurodegeneration in stressed ischemic animals.

In the ischemic brain, HMGB1 is released within hours from necrotic neurons and astrocytes [50,51,52], and later from activated microglia [53,54]. Consistent with these findings, at 7 and 14 days post-ischemia, we observed that HMGB1 was translocated from the nucleus to the cytoplasm in activated microglia, suggesting that these are the main cellular sources of HMGB1. HMGB1 cytoplasmic translocation was potentiated in stressed ischemic animals, showing the impact of chronic stress on microglial inflammatory response triggered by HMGB1 release following global ischemia. In line with our findings, Iwata et al. [55] reported that HMGB1 release was worsened by comorbid diabetes and aggravated ischemic brain injury in a rodent model of stroke. Kim et al. [17] also showed that HMGB1 exacerbates systemic and brain inflammation in a rodent model of post-stroke infection. The results of our study contribute to a growing literature suggesting that elevated circulating HMGB1 can be used as a biomarker associated with poor outcome in ischemic brain injury with comorbid conditions [17,18,55,56,57].

### 3.2. Effects of Stress Exposure Prior to Ischemia on NLRP3 Inflammasome Activation

The second important finding of our study was that comorbid stress primed the NLRP3 inflammasome to subsequent ischemia, upregulating its pathway activation in the hippocampus. This led to a greater increase in TLR4, P2X7, IκBα activation, NLRP3, ASC, cleaved caspase-1, and pro-inflammatory cytokine IL-1β levels in the Stress+ISCH group compared to the ischemia-alone group. Here, we confirmed the findings of Weber et al. [13] showing that stress-induced NLRP3 inflammasome priming resulted in increased NLRP3 levels but did not alter IL-1β production in the hippocampus. Other studies [24,58] have reported that, in addition to NLRP3 upregulation, stress increases hippocampal cleaved caspase-1 and IL-1β levels; but we did not observe this effect in our stressed controls. However, we did confirm that global ischemia activated the NLRP3 inflammasome pathway [25,26,27]. This observation supports previous studies showing that NLRP3 inflammasome inhibition reduces inflammation and ameliorates ischemic injury. In pigs, Qian et al. [27] observed a similar up-regulation in NLRP3, cleaved caspase-1 and IL-1β as early as 30 h after cardiac arrest. Similarly, Yang et al. [26] found an increase in NLRP3 and Caspase-1 at 72 h post-ischemia.

Accordingly, Thakkar et al. [25] reported that global ischemia robustly increased NLRP3 inflammasome pathway activation, including cleaved caspase-1 and IL-1β expression in the hippocampus, with peak levels observed at 7 days. In our study, NLRP3 inflammasome activation peaked at 14 days post-ischemia. In line with our results on comorbid stress combined with global ischemia, Hong et al. [59] found that comorbid diabetes remarkably amplifies NLRP3, caspase-1, and IL-1β expression in the ischemic core. Collectively, these data reinforce the idea that inflammation-related comorbidities such as comorbid stress and diabetes, drive NLRP3 inflammasome priming associated with worsening ischemic brain injury.

### 3.3. Effects of Stress Exposure Prior to Ischemia on Autophagy

In the present study, we observed no changes in serum LC3-II levels at 7 or 14 days post-ischemia, suggesting that ischemia induces peripheral autophagic changes that may not persist. In the ischemic brain, Tian et al. [60] detected LC3-positive autophagic cells, mostly neurons, and an increase in LC3-II expression, with a peak at day 1 post-ischemia, indicating that neuronal autophagy occurs early after stroke and further suggesting that astrocytes and microglia might be more resistant to ischemia-induced autophagy. Similarly, Hwang et al. [61] demonstrated that global ischemia activates autophagy in vulnerable hippocampal CA1 neurons destined to die. Huang et al. [62] also reported LC3-II an early increase in the neurons, which began at 6 h and peaked at 24 h. Zhu et al. [33] showed autophagy induction in hippocampal brain tissue from 6 h up to 48 h after cardiac arrest. Wang et al. [63] observed an increased LC3-II protein level after cardiac arrest; that peaked between 3 and 6 h and then gradually decreased by 24 h. Wei et al. [34] confirmed an early increase in LC3-II at 6 and 12 h within the cortex from mice subjected to cardiac arrest. However, Wu et al. [64] and Li et al. [65] observed lower levels of autophagy markers in the ischemic cortex compared to non-ischemic controls at days 7 and 14 post-ischemia. This is consistent with our observations of reduced hippocampal levels of LC3-II, indicating that autophagy was impaired at later time points after global ischemia when microglial activation peaks. This finding supports the beneficial effects of autophagy inducers in ischemic brain injury, although this concept remains controversial [66,67].

Next, we examined the impact of comorbid stress on the autophagic response to global ischemia. We found that 8-day social defeat stress induced a reduction in hippocampal LC3-II levels similar to the effects of global ischemia. Our data differ from those of Xiao et al. [68] who reported that 4-week, chronic, mild, unpredictable stress increased hippocampal LC3-II levels, suggesting that autophagic activity is elevated under conditions of stress. In contrast, Zhang et al. [69] showed that chronic restraint stress for a period of 21 days inhibited autophagy, indicated by reduced levels of autophagy markers such as LC3-II and p62. Similarly, Wang et al. [70] demonstrated that social isolation extended up to 6 months inhibits autophagy activation. It is important to note here that immune responses to stressors are highly dependent upon type, duration, and severity of stress [71]. Such differences in study designs may account for some of the discrepant findings. In our experiment, we found that the Stress+ISCH group showed lower hippocampal LC3-II levels than unstressed ischemic animals, suggesting that chronic stress, when followed by ischemia, leads to greater autophagy impairment.

We and others have also reported that microglia respond to stress by adopting a primed phenotype, which then exhibits an exacerbated inflammatory response to subsequent cerebral ischemia. Using an *in vitro* inflammasome assay [72], we confirmed that stimulation with LPS followed by ATP, a classical inflammasome activator, resulted in increased IL-1β production in primary microglia compared to LPS alone. Similar NLRP3-dependent inflammatory responses have been reported in primary microglia and mouse N13 and human HMC3 microglia cell lines [73,74,75]. We also found that microglia treated with LPS+ATP showed a reduced number of LC3-positive vesicles, indicating that NLRP3 inflammasome activation *in vitro* compromises autophagy in microglia. This is consistent with the report of Houtman et al. [29], who demonstrated, using super-resolution microscopy, that NLRP3 colocalizes with LC3-positive vesicles in microglia treated with LPS+ATP, indicating that autophagy modulates microglial pro-inflammatory cytokine production by degrading NLRP3.

In addition to studying the *in vitro* effects of NLRP3 inflammasome activation in primary microglia, we examined the effects of stress priming on IL-1β production and autophagy in hippocampal microglia isolated from stressed animals. As expected [49,76,77], we observed that stress-primed microglia showed a potentiated IL-1β production when stimulated *ex vivo* with LPS compared to unprimed microglia isolated from unstressed animals. Subsequently, we found that stress-primed microglia showed fewer LC3-positive vesicles than unprimed microglia. Taken together, the data suggest that, like LPS-primed microglia, stress-primed microglia are more reactive to a second insult and more susceptible to autophagy impairment, and that microglial priming induced by inflammation-related comorbidities substantially affects disease severity and outcome. We propose that autophagy induction in primed microglia can reduce the deleterious inflammatory responses in ischemic stroke with comorbid stress. Indeed, Jin et al. [31] showed that autophagy induction promotes microglial polarization toward the M2 protective phenotype and resolves inflammation, while autophagy inhibition aggravates the pro-inflammatory M1 phenotype leading to neurotoxicity.

### 3.4. PROG Treatment of Stress-Induced Microglial Priming

There are now over 400 publications in the literature showing that the pleiotropic, neurosteroid PROG and its metabolites are neuroprotective and functionally effective in various, pre-clinical models of brain injury [78,79,80]. Among its pleiotropic effects, PROG reduces pro-inflammatory cytokine production and regulates microglial activation and M1/M2 polarization in rodent models of ischemic brain injury [9,36,42,44,46,81]. In the present study, we showed that treatment with PROG reduced alarmin HMGB1 release and NLRP3-dependent inflammation, and enhanced autophagy (1) in primary microglia after NLRP3 inflammasome activation *in vitro*, (2) in stress-primed hippocampal microglia stimulated with LPS *ex vivo*, and (3) in a rodent model of global ischemia combined with social defeat stress. Taken together, our findings provide new insights into the molecular mechanisms underlying PROG’s protective effects on the microglial inflammatory response induced by ischemia and worsened by comorbid stress. First, our data demonstrated that PROG reduced stress- and ischemia-induced HMGB1 levels in the serum and in the brain. This immunomodulatory effect of PROG was previously shown in human endometrial tissue, where HMGB1 is presumably implicated in endometrium physiology and/or embryo development [82].

Second, we found that PROG attenuated NLRP3 pathway activation, as shown by a reduction in TLR4, P2X7, IκBα, NLRP3, ASC, caspase-1, and IL-1β hippocampal levels in PROG-treated animals compared to non-treated animals at 7 and 14 days post-ischemia. Our findings confirm and extend previous studies assessing the effects of PROG on pro-inflammatory cytokine production in LPS-stimulated BV-2 microglia [38,83] or on the NLRP3/ASC/caspase 1 axis after *in vitro* hypoxia [45]. In contrast, Lammerding et al. [35] surprisingly reported that NLRP3 and cleaved caspase-1 levels decrease, rather than increase, in the peri-infarct area, and that PROG reduces only ASC and IL-1β levels at 24 h after stroke. Furthermore, Aryanpour et al. [84] demonstrated that PROG switches microglia from the pro-inflammatory M1 phenotype to the protective M2 phenotype and suppresses NLRP3 inflammasome expression in a cuprizone-induced demyelination mouse model.

Third, we showed that PROG enhanced autophagy impaired by comorbid stress and global ischemia, as evidenced by higher LC3-II levels in both stressed and unstressed ischemic animals treated with PROG compared to non-treated animals. This is consistent with a larger number of LC3-positive vesicles observed in PROG-treated primary microglia stimulated with LPS+ATP *in vitro* and a reduced IL-1β production compared to untreated controls. Similar PROG effects were found in stress-primed hippocampal microglia stimulated with LPS *ex vivo*. In our experiments, PROG’s beneficial effects were blocked by 3-MA, an autophagy inhibitor, worsening microglial inflammatory response. These results are in accordance with the findings of Kim et al. [85] in cultured astrocytes exposed to neurosteroids. They showed that PROG induces the formation of autophagosomes and increases LC3-II levels. This group [86] further reported that PROG increases autophagic flux in a model of amyotrophic lateral sclerosis. Hong et al. [87] demonstrated that PROG reduces NLRP3-inflammasome activation via enhancing autophagy in astrocytes stimulated with LPS or amyloid-β oligomers.

Based on our findings and previous reports, we suggest that PROG could have modulated NLRP3-dependent inflammation and autophagy in microglia through multiple targets: (1) PROG is known to suppress pro-inflammatory cytokine production by inhibiting the TLR4/NF-κB pathway [81,83]; (2) PROG reduces NLRP3 pathway activation and the expression of inflammasome components [35,45,84]; and (3) PROG induces autophagy in astrocytes [85,86,87]. In line with Mir et al. [88], we speculate that PROG may exert pro-autophagic effects through its membrane receptor PGRMC1, as PGRMC1 binds to LC3-II and promotes autophagy in human cell lines. PROG also suppresses mTOR, a negative regulator of autophagy, as shown in a rodent model of traumatic brain injury [89] and human glioblastoma cells [90,91]. With its multiple pleiotropic effects, PROG offers greater therapeutic potential compared to single-target agents to treat stroke patients with inflammation-related comorbid conditions.

## 4. Materials and Methods

### 4.1. Animals

Male Sprague-Dawley rats (250–275 g, Charles River) were individually housed in a climate-controlled room under a 12:12-h reverse light/dark cycle (lights off at 11:00 h), and food and water were provided ad libitum throughout the study. All animal procedures were carried out according to the NIH laboratory care standards and have been approved by the Institutional Animal Care and Use Committee of Emory University (protocol DAR-2003279, 21 December 2018). The experiments reported here were in accordance with the ARRIVE guidelines.

### 4.2. Experimental Groups and In Vivo Treatment

After 7 days of acclimatization, animals were randomly assigned to the following treatment groups: (1) Sham (*n* = 24), unstressed controls given vehicle; (2) Sham+PROG (*n* = 24), unstressed controls given PROG; (3) Stress+Sham (*n* = 24), stressed controls given vehicle; (4) Stress+Sham+PROG (*n* = 24), stressed controls given PROG; (5) ISCH (*n* = 24), unstressed animals subjected to global ischemia given vehicle; (6) ISCH+PROG (*n* = 24), unstressed animals subjected to global ischemia given PROG; (7) Stress+ISCH (*n* = 24), stressed animals subjected to global ischemia given vehicle; and (8) Stress+ISCH+PROG (*n* = 24), stressed animals subjected to global ischemia given PROG. PROG (8 mg/kg/b.w.; P3972, Sigma, St. Louis, MO, USA) or vehicle (20% 2-hydroxypropyl b-cyclodextrin in sterile water; H107, Sigma, St. Louis, MO, USA) were administered by intraperitoneal injection at 2 h post post-ischemia followed by subcutaneous injections at 6 h, and once every 24 h post-injury for 5 days, and then for 2 days with progressively halved dosages. This dose of PROG has effectively produced functional improvements and reduced ischemic injury severity [92,93]. PROG and vehicle were prepared and coded in advance, thus that investigators administering these solutions were blinded to their identity.

### 4.3. Social Defeat Stress

We used the social defeat paradigm to model social stress in male rats [94,95]. Social defeat has been considered as a valid stressor in humans [96,97]. Animals were placed in the cage of an older, aggressive, dominant male Long-Evans rat (retired breeders, Charles River), with 5 min of physical interaction, and followed by 25 min of threat, for 8 consecutive days. Animals assigned to the unstressed groups remained undisturbed in their home cages.

### 4.4. Global Cerebral Ischemia

Once the stress period ended, animals were subjected to global ischemia by the 4-vessel occlusion model [98,99]. Briefly, under isoflurane anesthesia, the vertebral arteries were permanently occluded by electrocauterization, and 48 h later, the common carotid arteries were transiently occluded for 8 min using microvascular clamps. This type of ischemic insult induced a selective neuronal loss in the hippocampal CA1 region of rodents [100] and humans [101]. Sham-operated animals used as controls underwent the same anesthetic and surgical procedures as ischemic animals, except occlusion of the common carotid arteries.

### 4.5. Tissue Collection

At days 7 and 14 after surgery, the rats were deeply anesthetized with isoflurane for euthanasia, a cardiac blood sample was collected, and then all groups were divided into 2 subgroups: One for histological assessments and other for biochemical analysis. In subgroup 1, animals were intracardially perfused with cold 1xPBS (46-013-CM, Corning, NY, USA) followed by 10% buffered formalin phosphate (SF100-4, Fisher Chemical, Corning, NY, USA), their brains were removed, and coronal brain slices containing the dorsal hippocampus were cryoprotected with 30% sucrose (S0389, Sigma, St. Louis, MO, USA) and then frozen. Slices were cut into 10 µm sections, which were mounted on slides and stored at −20 °C. In subgroup 2, rats were decapitated, and their brains quickly removed. Hippocampi were dissected, rapidly frozen, and stored at −80 °C.

### 4.6. Serum Measurements

Cardiac blood was centrifuged at 2000× *g* for 25 min at 4 °C, and serum was collected and analyzed using a commercial ELISA kit for HMGB1 (LS-F4039, LifeSpan Biosciences, Inc., Seattle, WA, USA) and LC3B (LS-F19802, LifeSpan Biosciences, Inc., Seattle, WA, USA) as per manufacturer’s instructions.

### 4.7. Immunohistochemistry

Brain sections were permeabilized with 0.3% Triton X-100 (T8787, Sigma, St. Louis, MO, USA) for 10 min, blocked with 3% bovine serum albumin (BSA, A3294, Sigma, St. Louis, MO, USA) for 1 h at room temperature, and then incubated overnight at 4 °C with a primary antibody anti-HMGB1 in combination with cell-specific markers anti-NeuN for neurons, anti-GFAP for astrocytes, or anti-CD11b for microglia (Table 1). After washing, sections were incubated with the corresponding secondary antibodies (Table 1) for 1 h at room temperature. Next, sections were mounted with Vectashield antifade mounting medium with DAPI (H-1200, Vector Laboratories, Burlingame, CA, USA). Fluorescence images of the whole dorsal hippocampus were acquired using the VS120 virtual microscopy slide scanning system (Olympus).

### 4.8. Western Blotting

Isolated hippocampi were homogenized in T-PER buffer (78510, Thermo Fisher Scientific, Waltham, MA USA) containing a protease inhibitor cocktail (P8340, Sigma, St. Louis, MO, USA), and then centrifuged at 10,000× *g* for 10 min at 4 °C. Supernatant was collected, and protein concentration was determined by Bradford assay (1856210, Thermo Fisher Scientific, Waltham, MA, USA). Forty micrograms of protein for each sample were separated on SDS-polyacrylamide gels, transferred to a PVDF membrane, and then blocked with 5% nonfat milk or BSA for 1 h at room temperature. Membranes were incubated with primary antibodies to assess NLRP3 signaling pathway and autophagic flux (Table 1) overnight at 4 °C. After washing, membranes were incubated with the corresponding secondary antibodies (Table 1) for 1 h at room temperature. Blots were developed using a chemiluminescent horseradish peroxidase antibody detection reagent (E2400, Denville Scientific, Inc., Holliston, MA, USA) for 2 min. Chemiluminescent bands were detected on an autoradiography film in a darkroom, and their densities were measured using NIH ImageJ FIJI software. Protein levels were normalized to β-actin, which was used as a loading control. Data were represented as the fold change in protein levels relative to the Sham group. The mean ± SEM was calculated for each group (*n* = 6).

### 4.9. In Vitro Inflammasome Assay

Mixed glial cultures were prepared from cerebral cortices of postnatal day 1–2 C57BL/6J male mouse pups (Charles River), as described by Bronstein et al. [102]. After mechanical dissociation and treatment with trypsin/EDTA solution (SV30031.01, HyClone, Marlborough, MA, USA), cortical cells were seeded in DMEM medium (10-013-CV, Corning, NY, USA) supplemented with 10% FBS (97068-085, VWR Life Science Seradigm, Radnor, PA, USA) and antibiotic-antimycotic solution (containing 10,000 U/mL penicillin, 10,000 µg/mL streptomycin, and 25 µg/mL Amphotericin B, 15240-096, Thermo Fisher Scientific, Waltham, MA, USA), and cultured at 37 °C and 5% CO_2_. Once confluent, mixed glial cultures were subjected to mild trypsinization with trypsin/EDTA solution in a serum-free DMEM medium to isolate microglia, according to Saura et al. [103]. Microglia were plated in 96-well plates (CC7682-7596, CytoOne, Ocala, FL, USA) previously coated with poly-D-lysine (PDL, P0899, Sigma, St. Louis, MO, USA) and allowed to adhere overnight. To activate the NLRP3 inflammasome, microglia were primed with 1 µg/mL LPS (*E. coli* 0111:B4, InvivoGen, San Diego, CA, USA) for 2 h followed by stimulation with 5 mM ATP (A6419, Sigma) for 1 h [72], and concomitantly treated with 20 µM PROG (P3972, Sigma, St. Louis, MO, USA) dissolved in dimethyl sulfoxide (DMSO, BP231-1, Thermo Fisher Scientific, Waltham, MA, USA) as previously reported [42]. Autophagy was inhibited with 5 mM 3-methyladenine (3-MA, ab120841, Abcam, Cambridge, MA, USA) given 2 h prior to LPS. Cell supernatants were harvested and IL-1β levels were measured using the mouse IL-1β ELISA kit (DY401-05, R&D Systems, Minneapolis, MN, USA). *In vitro* assays were performed in triplicate.

### 4.10. Neuroinflammatory Priming to Stress

Hippocampal microglia were isolated from stressed and unstressed rats as described by Frank et al. [104]. Following the last social defeat stress exposure, the rats were fatally anesthetized with isoflurane and intracardially perfused with cold 1xPBS, their brains were rapidly extracted, and hippocampi dissected and homogenized on ice. Hippocampal homogenates were filtered through a 40 μm cell strainer (352340, Falcon, Corning, NY, USA) and centrifuged at 1200× *g* for 45 min at 20 °C using a 0/50/70% Percoll (P4937, Sigma, St. Louis, MO, USA) gradient. After centrifugation, microglial cells were extracted from the 50/70% Percoll interface, washed and resuspended in DMEM medium with 10% FBS. Microglia were seeded in 96-well plates, allowed to adhere, and then challenged *ex vivo* with 10 ng/mL LPS for 18 h [79], and concomitantly treated with 20 µM PROG dissolved in DMSO. Autophagy was inhibited with 5 mM 3-MA given 2 h prior to LPS. Cell supernatants were harvested, and IL-1β levels were measured using the rat IL-1β ELISA kit (DY501-05, R&D Systems, Minneapolis, MN, USA). *Ex vivo* experiments were performed in triplicate.

### 4.11. Immunocytochemistry

Microglia were plated in chamber slides (154526, Lab-Tek II, Sigma, St. Louis, MO, USA) previously coated with PDL and treated as described above. After treatments, cells were fixed with 10% formalin for 10 min, then stained with a LC3 antibody overnight at 4 °C, followed by secondary antibody staining (Table 1) for 1 h at room temperature. Nuclei were stained with DAPI (D9542, Sigma, St. Louis, MO, USA). Slides were then mounted with Fluorescence Mounting Medium (H-1000, Vector Laboratories, Burlingame, CA, USA) and examined using a 60X TIRF oil objective on a Nikon A1R HD25 confocal microscope. Images were acquired with the NIS-Elements C Imaging software. The number of LC3-positive vesicles per cell was quantified using NIH ImageJ FIJI software. A threshold was set for pixel size with the GDSC plugin and then applied to images for extracting the cytosolic vesicles from the background. The number of LC3-positive vesicles was counted with the 3D Objects Counter plugin in >20 different cells randomly selected for each condition. Cell counts were averaged to provide a single value for each condition.

### 4.12. Statistical Analyses

A power analysis determined a sample size of 6 animals per group to detect an effect size of ≥0.50 with a power of 0.8 at α = 0.05. All data were expressed as mean ± SEM. Data were analyzed using one-way ANOVA followed by Tukey’s post-hoc test. Calculations were obtained using Prism V 6.0 (GraphPad Software Inc.). We reported p-values according to the latest guidelines of the American Statistical Association [105].

## 5. Conclusions

In conclusion, our *in vivo*, *in vitro* and *ex vivo* data confirm and extend findings on the impact of comorbid stress on worsening the inflammatory response to ischemia. Our findings suggest that chronic stress can drive microglia to adopt a primed phenotype. After an ischemic insult, the stress helps to produce an exaggerated inflammatory response associated with increased HMGB1 release, NLRP3 inflammasome overactivation, and autophagy impairment. PROG modulates stress-induced microglial priming as one of the molecular mechanisms by which this neuroprotective steroid ameliorates inflammation and ischemic brain injury. To complete this picture, we recognize the need for assessing the expression level of membrane progesterone receptors in microglia to obtain direct evidence of progesterone’s effects on microglial response. Such experiments will help determine progesterone’s multiple effects are primarily mediated through this receptor. Additional mechanistic studies might also investigate the role of the membrane progesterone receptor in microglial HMGB1 distribution, NLRP3 signaling activation and autophagic response to injury. However, we recognize the need for assessing the expression level of membrane progesterone receptor in microglia to obtain direct evidence of progesterone’s effects on microglial response are primarily mediated through this receptor. Further detailed mechanistic studies to investigate the role of membrane progesterone receptor in microglial HMGB1 distribution, NLRP3 signaling activation and autophagic response are also needed. Targeting membrane progesterone receptor can be done by using either pharmacological inhibitors or genetical methods.

## Figures and Tables

**Table 1:** Antibodies used for immunofluorescence/Western blotting.

Antibody	Host	Dilution	Catalog Number	Vendor
**(A) Primary antibodies**				
HMGB1	rabbit	1:100/1:1000	ab18256	Abcam
NeuN	mouse	1:1000	MAB377	Chemicon
GFAP	rat	1:1000	13-0300	Invitrogen
CD11b	mouse	1:100	MCA275R	AbD serotec
TLR4	mouse	1:500	76B357.1	ThermoFisher
P2X7	mouse	1:100	sc-514962	Santa Cruz Biotechnology
NLRP3	rabbit	1:1000	ab214185	Abcam
IκBα	rabbit	1:1000	9242	Cell Signaling
Phospho-IκBα	mouse	1:1000	9246	Cell Signaling
ASC	mouse	1:100	sc-514414	Santa Cruz Biotechnology
Caspase-1	mouse	1:100	sc-398715	Santa Cruz Biotechnology
IL-1β	rabbit	1:500	ab9787	Abcam
LC3	rabbit	1:100/1:1000	12741	Cell Signaling
β-actin	mouse	1:10,000	A5316	Sigma
**(B) Secondary antibodies**				
Alexa Fluor 488 anti-rabbit	donkey	1:200	A21206	Invitrogen
Alexa Fluor 568 anti-mouse	goat	1:200	A11004	Invitrogen
Alexa Fluor 594 anti-rat	goat	1:200	A11007	Invitrogen
Anti-mouse	goat	1:2000	074-1806	KPL
Anti-rabbit	goat	1:2000	074-1506	KPL

## Supplementary Materials

Supplementary materials can be found at https://www.mdpi.com/1422-0067/21/11/3740/s1.

## Abbreviations

ATP	Adenosine triphosphate
ASC	Apoptosis-associated speck-like protein containing a caspase recruitment domain
BSA	Bovine serum albumin
DMSO	Dimethyl sulfoxide
HMGB1	High-mobility group box-1
IκBα	Nuclear factor of kappa light polypeptide gene enhancer in B-cells inhibitor, alpha
IL	Interleukin
LC3	Microtubule-associated protein light chain 3
LPS	Lipopolysaccharide
mTOR	Mammalian target of rapamycin
3-MA	3-methyladenine
NF-κB	Nuclear factor of kappa light polypeptide gene enhancer in B-cells
NLRP3	NOD-like receptor pyrin domain containing 3
P2X7R	Purinergic receptor P2X, ligand gated ion channel, 7
PROG	Progesterone
PGRMC1	Progesterone-associated membrane component 1
TLR4	Toll-like receptor-4
