# Supplementary material for: Progesterone Attenuates Stress-Induced NLRP3 Inflammasome Activation and Enhances Autophagy Following Ischemic Brain Injury
Source: Int J Mol Sci. 2020 May 26;21(11):3740. doi: 10.3390/ijms21113740 (PMC7312827; doi:10.3390/ijms21113740)
Supplement: Supplementary file 1 [file ijms-21-03740-s001.pdf]

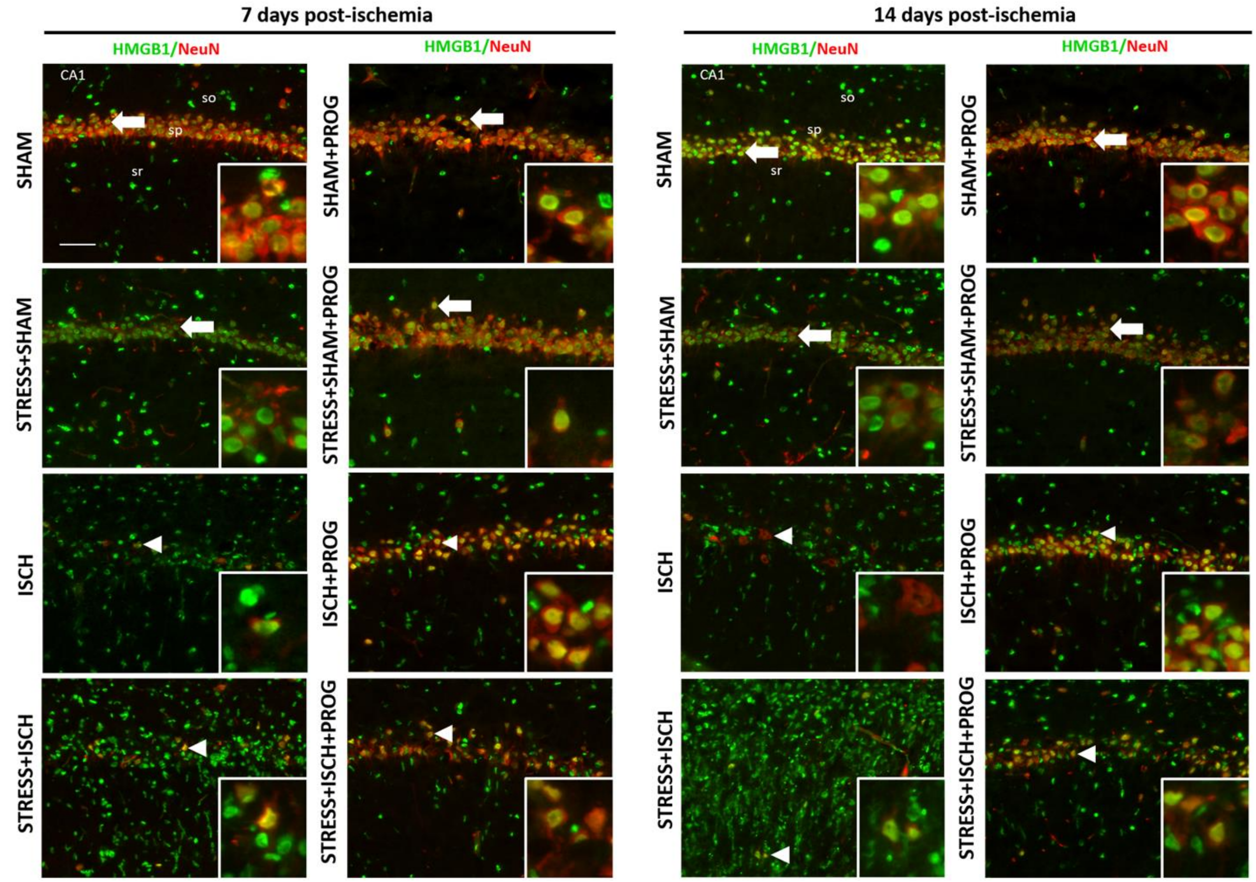

Supplementary Figure 1. Effects of stress and progesterone on HMGB1 distribution in neurons. Representative immunofluorescence merged images showing the distribution of HMGB1 (green) and NeuN positive neurons (red) in the hippocampal CA1 region at 7 and 14 days after global ischemia, or from sham animals. White arrows indicate HMGB1 is located in the nucleus of neurons. so, stratum oriens; sp, stratum pyramidale; sr, stratum radiatum. Scale bar: 50 μm.

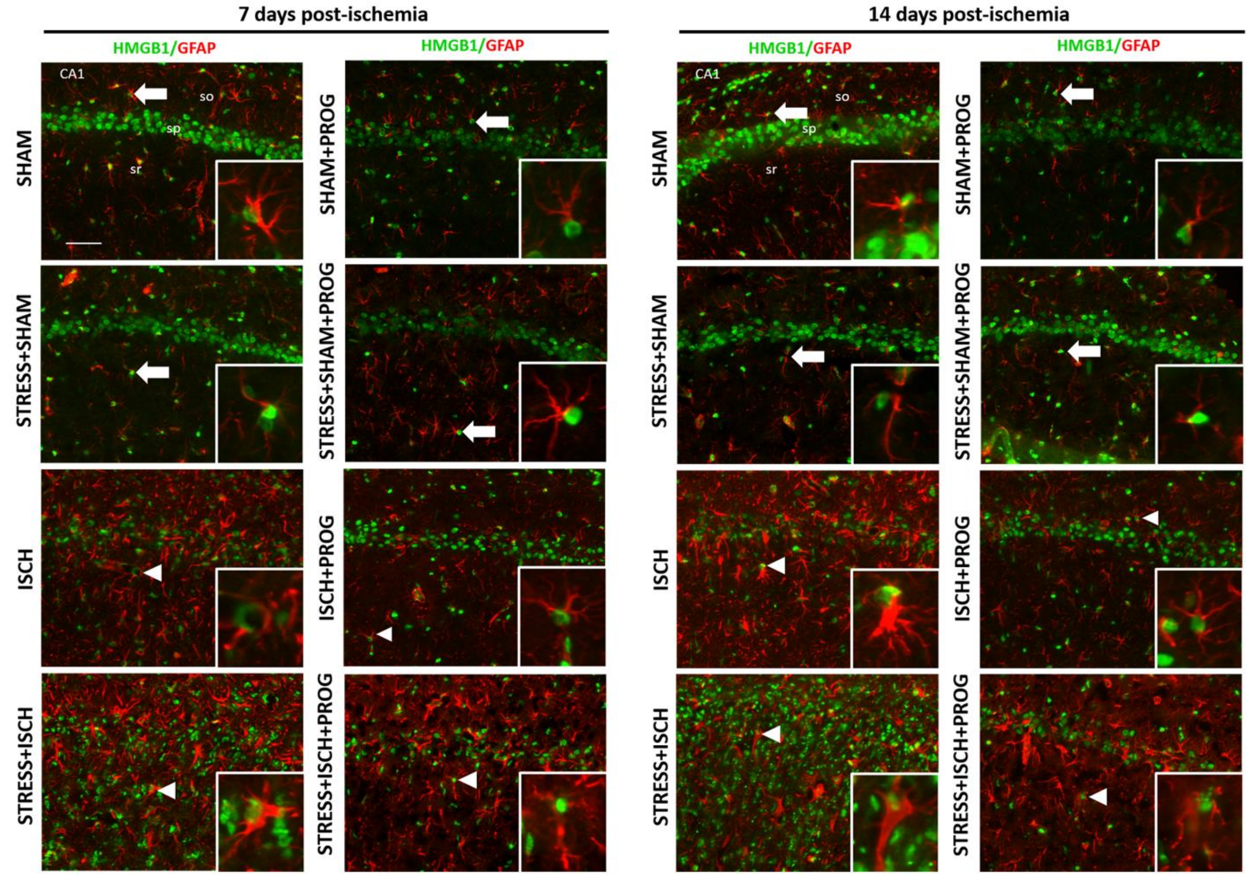

Supplementary Figure 2. Effects of stress and progesterone on HMGB1 distribution in astrocytes. Representative immunofluorescence merged images showing the distribution of HMGB1 (green) and GFAP positive astrocytes (red) in the hippocampal CA1 region at 7 and 14 days after global ischemia, or from sham animals. White arrows indicate HMGB1 is located in the nucleus of astrocytes. so, stratum oriens; sp, stratum pyramidale; sr, stratum radiatum. Scale bar: 50 μm.
